# Association between atherogenic risk-modulating proteins and endothelium-dependent flow-mediated dilation in coronary artery disease patients

**DOI:** 10.1007/s00421-022-05040-z

**Published:** 2022-10-28

**Authors:** Andrea Tryfonos, Joseph Mills, Daniel J. Green, Anton J. M. Wagenmakers, Ellen A. Dawson, Matthew Cocks

**Affiliations:** 1 Research Institute for Sport and Exercise Science, Liverpool John Moores University, Liverpool, L3 3AF UK; 2 Department of Life Science, European Cyprus University, 2404 Nicosia, Cyprus; 3 Liverpool Heart and Chest Hospital, Liverpool, L14 3PE UK; 4 School of Human Sciences (Exercise and Sport Science), The University of Western Australia, Crawley, WA 6009 Australia

**Keywords:** Atherosclerosis, Coronary artery disease, Endothelial cells, Endothelial dysfunction, Exercise-induced dilation, Flow-mediated dilation

## Abstract

**Purpose:**

Endothelial dysfunction is an early and integral event in the development of atherosclerosis and coronary artery disease (CAD). Reduced NO bioavailability, oxidative stress, vasoconstriction, inflammation and senescence are all implicated in endothelial dysfunction. However, there are limited data examining associations between these pathways and direct in vivo bioassay measures of endothelial function in CAD patients. This study aimed to examine the relationships between in vivo measures of vascular function and the expression of atherogenic risk-modulating proteins in endothelial cells (ECs) isolated from the radial artery of CAD patients.

**Methods:**

Fifty-six patients with established CAD underwent trans-radial catheterization. Prior to catheterization, radial artery vascular function was assessed using a) flow-mediated dilation (FMD), and b) exercise-induced dilation in response to handgrip (HE%). Freshly isolated ECs were obtained from the radial artery during catheterization and protein content of eNOS, NAD(P)H oxidase subunit NOX2, NFκB, ET-1 and the senescence markers p53, p21 and p16 were evaluated alongside nitrotyrosine abundance and eNOS Ser^1177^ phosphorylation.

**Results:**

FMD was positively associated with eNOS Ser^1177^ phosphorylation (*r* = 0.290, *P* = 0.037), and protein content of p21 (*r* = 0.307, *P* = 0.027) and p16 (*r* = 0.426, *P* = 0.002). No associations were found between FMD and markers of oxidative stress, vasoconstriction or inflammation. In contrast to FMD, HE% was not associated with any of the EC proteins.

**Conclusion:**

These data revealed a difference in the regulation of endothelium-dependent vasodilation measured in vivo between patients with CAD compared to previously reported data in subjects without a clinical diagnosis, suggesting that eNOS Ser^1177^ phosphorylation may be the key to maintain vasodilation in CAD patients.

## Introduction

Coronary artery disease (CAD) is a leading cause of death in the Western world with over 370,000 deaths annually (Mozaffarian et al. 2016). Atherosclerosis is proposed as the underlying cause of CAD (Ross 1993). In this context, endothelial (dys)function has emerged as an early and integral event in the atherogenic process (Davignon and Ganz 2004; Thijssen et al. 2019). Indeed, markers of endothelial function are typically lower in CAD patients compared to aged-matched controls (Oz et al. 2012) and worsen with the progression of CAD (Manganaro et al. 2014).

Endothelial dysfunction is often characterized by a reduction in nitric oxide (NO) bioavailability. NO bioavailability is determined by the balance between NO synthesis and scavenging by superoxide anions and related reactive oxygen species. It is hypothesized that increased production of reactive oxygen species (Elahi et al. 2009) plays a major role in reduced NO bioavailability within CAD patients (Cai and Harrison 2000; Ohara et al. 1993). In support of this, elevated markers of oxidative stress have been associated with coronary endothelial dysfunction in CAD patients (Lavi et al. 2008). Furthermore, mRNA expression of NOX2, the catalytic subunit of the NAD(P)H oxidase complex, was elevated in the coronary arteries of CAD patients compared to those without CAD (Guzik et al. 2006). Age-associated reductions in endothelial function are also associated with markers of oxidative stress [NADPH oxidase-p47^phox^ (Donato et al. 2009) and nitrotyrosine (Donato et al. 2007)].

In contrast, coronary endothelial dysfunction was not associated with reduced basal NO production in CAD patients (Lavi et al. 2008). In addition, age-associated reductions in endothelium-dependent dilation were not associated with eNOS protein content or eNOS Ser^1177^ phosphorylation (Donato et al. 2007; Pierce et al. 2011). Indeed, NO production was increased in hypercholesterolemic rabbits despite the severely impaired endothelium-dependent dilation (Minor et al. 1990). This suggests that signaling pathways leading to eNOS activation remained intact. In summary, the above suggests a causal role for increased NO scavenging in the development of endothelial dysfunction and CAD. Conversely, reduced NO production appears unlikely to play a major role in the development of CAD.

Increased reactive oxygen species production may also stimulate the expression of other pro-atherogenic factors. Endothelin 1 (ET-1) (Lerman et al. 1991; Donato et al. 2009) and nuclear factor κB (NFκB) (Cominacini et al. 2005; Real et al. 2010; Silver et al. 2007; Donato et al. 2007) were elevated in CAD patients and/or subjects with an atherosclerotic-prone phenotype. Endothelial ET-1 expression is associated with reduced endothelium-dependent dilation in aging (Donato et al. 2007, 2009), suggesting its involvement in endothelial dysfunction.

A pro-oxidant and -inflammatory *milieu* may trigger cellular senescence in the vasculature (Katsuumi et al. 2018; Campisi and d'Adda di Fagagna 2007; Childs et al. 2017). Cellular senescence is a stress response resulting in irreversible growth arrest of a cell. Senescence has emerged as a potential driver for endothelial dysfunction (Donato et al. 2015) and atherosclerotic diseases (Katsuumi et al. 2018). Although there is limited evidence toward endothelial cell (EC) senescence and endothelial (dys)function, the expression of senescence markers (p53, p21 and p16) was inversely associated with endothelium-dependent dilation in older humans and mice (Bhayadia et al. 2016; Rossman et al. 2017). These studies may suggest the potential role of senescent phenotype in endothelial dysfunction and subsequent development of atherosclerosis.

All the above demonstrate a complex vicious cycle with multiple interactions between markers of oxidative stress, inflammation, vasoconstriction and cellular senescence. All these directly and/or indirectly have an impact on NO bioavailability and thereby contribute to the development of CAD. However, how the changes in these atherogenic-modulating proteins influence the progression of endothelial dysfunction in patients with established CAD has limited direct evidence.

Most studies have used flow-mediated dilation (FMD) to assess in vivo endothelial function in humans. However, recent data from our team have shown impaired FMD but preserved dilation in response to localized exercise in CAD patients (Tryfonos et al. 2020). As such, these tests may reflect different components of vascular function. Hyperemic response to exercise represents an integrated response involving autonomic activity and numerous endothelium- and non-endothelium-dependent vasoactive systems (Hellsten et al. 2012). Importantly, exercise-induced dilation demonstrates a redundancy where there is an ability to compensate for an impaired pathway with alternative mechanisms (Hellsten et al. 2012). Assessing multiple pathways using distinct stimuli provides important information when presented alongside measurement of FMD (Nyberg et al. 2012). It is currently unknown whether exercise-induced dilation in CAD patients is associated with changes in the expression of atherogenic-modulating proteins.

Therefore, the primary aim of this study was to determine whether the endothelial expression of atherogenic-modulating proteins can predict FMD and/or exercise vasomotor responses in patients with established CAD. We hypothesized that both FMD and exercise-induced dilation would be negatively associated with the expression of proteins related to NO scavenging, inflammation, vasoconstriction and senescence. In contrast, there would be no relation between these tests and eNOS protein content or Ser^1177^ phosphorylation as markers of NO production.

## Methods

### Ethical approval

All participants provided written informed consent, and the study was approved by the Liverpool East NHS Research Ethics Committee (approval reference no. 13/NW/0088) and conformed to the Declaration of Helsinki.

### Participants

Sixty-four patients undergoing prospective percutaneous transluminal coronary angiography (PTCA) and/or percutaneous coronary intervention (PCI; angioplasty) were recruited from Liverpool Heart and Chest Hospital (LHCH). Patients were excluded if they were unable to give informed consent or had undergone a trans-radial cardiac catheterization or acute coronary syndrome within the last 3 months. Eight patients had angiographically normal coronaries following PTCA and were retrospectively excluded. As such, data from 56 patients with established CAD were included in this study. A summary of patient characteristics, medications, hemodynamic variables, and previous catheterization is included in Table 1.

**Table 1:** Characteristics of the study population (*n* = 56)

Clinical characteristic	Mean ± SD or *n* (%)
Age (years)	67 ± 9
Sex (males)	47 (84)
BMI (kg.m^**−2**^)	29.6 ± 5.3
Mean Blood Pressure (mmHg)	100 ± 11
Heart rate (beats/min)	61 ± 10
Previous trans-radial catheterization (PTCA and/or PCI)	23 (41)
Previous CABG	5 (9)
Previous MI > 3 months	19 (34)
Risk Factors	
Diabetes	17 (30)
Hypertension	34 (61)
Hypercholesterolemia	42 (75)
Current smoker	6 (11)
Ex-smoker	34 (61)
Positive family history	39 (70)
Medications	
Aspirin	50 (89)
Clopidogrel	6 (11)
Beta-Blocker	39 (70)
ACEI/ARB	31 (55)
Nitrate	44 (79)
Statin	46 (82)
Calcium-Blocker	17 (30.4)
Diuretics	8 (14.3)

*BMI* body mass index, *PTCA* percutaneous transluminal coronary angiography, *PCI* percutaneous coronary intervention, *CABG* coronary artery bypass graft, *MI* myocardial infarction, *ACEI* angiotensin−converting enzyme inhibitor, *ARB* angiotensin receptor blocker

### Study design

Vascular function measurements were assessed prior to catheterization (1–4 h). In brief, the catheterized radial artery was assessed using two non-invasive measurements: (a) flow-mediated dilation (FMD) followed by (b) vascular dilation to incremental handgrip exercise. The order of these tests was maintained in all participants. A standardized period of 10 min was observed between tests to allow artery function to recover to baseline levels. Patients then underwent PTCA and/or PCI and ECs were collected from the catheterized radial artery, as described below.

### Trans-radial cardiac catheterization and EC collection

PTCA and/or PCI were performed predominantly via the right radial artery under local anesthesia, as previously described (Tryfonos et al. 2020). Briefly, the catheterized artery was punctured with a 21-gage needle and a 5-7F hydrophilic sheath introducer (PreludeEase, MeritMedical, UK) was inserted. All patients received a weight-adjusted dose of heparin. Thirty patients received an additional ~ 1.5 mg/ml of vasodilator isosorbide di-nitrate, as indicated by the interventional cardiologist. Once the procedure was finished, the J-shaped guidewire (3 mm J TEF 150 cm × 0.35″, KIMAL, UK) was transferred immediately to ice-cold dissociation solution (~ 30 ml) (0.5% bovine serum albumin, 2 mmol/L EDTA, and 100 ug/mL heparin in Dulbecco's Phosphate-Buffered Saline (DPBS)). An additional sterile J-shaped guidewire was advanced into the radial artery (~ 3–4 cm above the sheath), run back and forth to collect ECs and transferred immediately to the dissociation buffer. All introducer sheaths were removed at the end of the procedure.

### Vascular function assessment

Vascular function measurements were completed in a quiet room between 0800 and 1100 h. Patients were fasted (including caffeine and alcohol) and asked to abstain from exercise and cigarettes for 12 h before the visit (Thijssen et al. 2019, 2011). Part of the vascular data (33 patients) has been published elsewhere (Tryfonos et al. 2020), where the methods have been described in detail. Briefly, patients rested in the supine position for > 10 min before blood pressure (BP) and heart rate (HR) were measured using an automated sphygmomanometer (GE Pro 300V2, Dinamap, Tampa, FL, USA). FMD and arterial response to exercise were then measured in the catheterized radial artery (10–15 cm proximal from the scaphoid bone in the wrist), using a 12-MHz multi-frequency linear array probe attached to a high-resolution ultrasound machine (T3000; Terason, Burlington, MA, USA) (Tryfonos et al. 2020). For the exercise protocol, patients performed a 3 × 3-min bout of handgrip exercise, in a seated position, at 5%, 10% and 15% of their pre-determined maximal voluntary contraction (MVC) (Takei 5420 Grip-D Digital Hand Grip Dynamometer, Japan), with 1-min rest between bouts. Arterial diameter/velocity recordings were measured at baseline and during the 1-min rest. The percentage difference in diameter from the baseline to peak in response to handgrip exercise (the maximum value at either 5%, 10% or 15% MVC) was then calculated (HE%) and used for further analysis. Custom-designed edge-detection and wall-tracking software was used to analyze both the FMD and handgrip exercise to minimize investigator bias (Woodman et al. 2001; Thijssen et al. 2011).

### Immunofluorescence microscopy

EC isolation and protein expression measurements were performed as previously described (Pierce et al. 2011; Donato et al. 2007). Briefly, ECs were recovered from the two J-shaped guidewires by washing and centrifugation. Cells were then fixed with 3.7% formaldehyde, plated on glass slides (VWR, REF number: 631-0705, USA) and frozen at − 80 °C until analysis. At the time of analysis, cells were re-hydrated and incubated for 1 h at room temperature with primary antibodies against eNOS (610,297, BD, USA), Phospho-eNOS Ser^1177^ (peNOS Ser^1177^) (07-428-I, Merck), NAD(P)H oxidase subunit NOX2 (kind gift from Prof Mark Quinn, Montana State University), tumor suppressor protein p53 (p53) (Ab26, Abcam, UK), cyclin-dependent kinase inhibitor 1 (p21) (Ab109520, Abcam, UK), cyclin-dependent kinase inhibitor 2A (p16) (Abc51243, Abcam, UK), nitrotyrosine (Ab7048, Abcam, UK), ET-1 (PA3-067, Thermo Fisher Scientific, USA), NFκB p65 (NB100-56,712, Novus, UK). Cells were also stained for vascular–endothelial (VE) cadherin (NB600-1409, Novus, UK) for positive identification of the endothelial phenotype and DAPI for nuclear integrity.

### Image capture

Images were acquired using an inverted confocal microscope (Zeiss LSM-710, Carl Zeiss, Germany) with a 63 × 1.3NA oil immersion objective. Positive staining for VE cadherin coupled with a single intact nucleus was used to reliably select ECs (Casey et al. 2017). DAPI was excited using the 405 nm line of the diode laser and detected with 371–422 nm emission. Alexa Fluor 488 was excited with the 488 nm line of the argon laser and detected with 493–559 nm emission. Alexa Fluor 546 and 633 fluorophores were excited with 543 nm and 633 nm lines of the helium–neon laser and 548–623 nm and 638–747 nm emission, respectively. The images were acquired at a resolution of 1,024 X 1,024 pixels and stored in 24-bit tagged image format file format.

### Image analysis

All images were analyzed using ImagePro Plus 5.1 (Media Cybernetics Inc, Bethesda, MD, USA). To ensure only the cytosolic fraction was assessed, nuclear regions of the ECs, identified through the DAPI stain, were extracted from the rest of the EC image identified using the VE cadherin image. The resulting mask was then overlaid onto the corresponding protein of interest image. Mean fluorescence intensity of the protein of interest signal was then quantified within the endothelial cytosolic-specific area. EC protein expression data are reported as ratios to human umbilical vein EC (HUVEC) protein expression. Slides were systematically scanned and at least 25 consecutive ECs were captured and analyzed for each protein in each patient, as previously suggested (Colombo et al. 2002). A single technician analyzed each batch of slides. Technicians were blinded to subject identity during the staining and analysis procedures.

### Statistical analysis

All statistical analyses were performed using IBM SPSS statistics for Windows, version 25.0. (Armonk, NY: IBM Corp). Pearson’s correlation analysis was used to determine relations of interest. In addition, hierarchical multiple regression models were performed when statistically significant Pearson’s correlations revealed between protein expression and in vivo vascular responses (FMD and/or HE%), to further examine whether patients’ characteristics (sex, age, BMI, and/or MAP) explain such associations. Data are presented as mean ± SD and statistical significance for all analyses was set at *P* ≤ 0.05.

## Results

Out of 56 patients, FMD was performed in 54 patients (2 × equipment failure) and HE data were available in 46 patients (2 × equipment failure, 1 × avoid exercise due to dizziness following FMD, 2 × previous injury to their hand, 1 × lack of time due to catheterization preparation, 4 × quality of images). In addition, EC samples were not taken from 2 patients (2 × complications during catheterization).

### Relationship between EC protein expression with endothelium-dependent dilation and exercise response

**Fig. 1:**
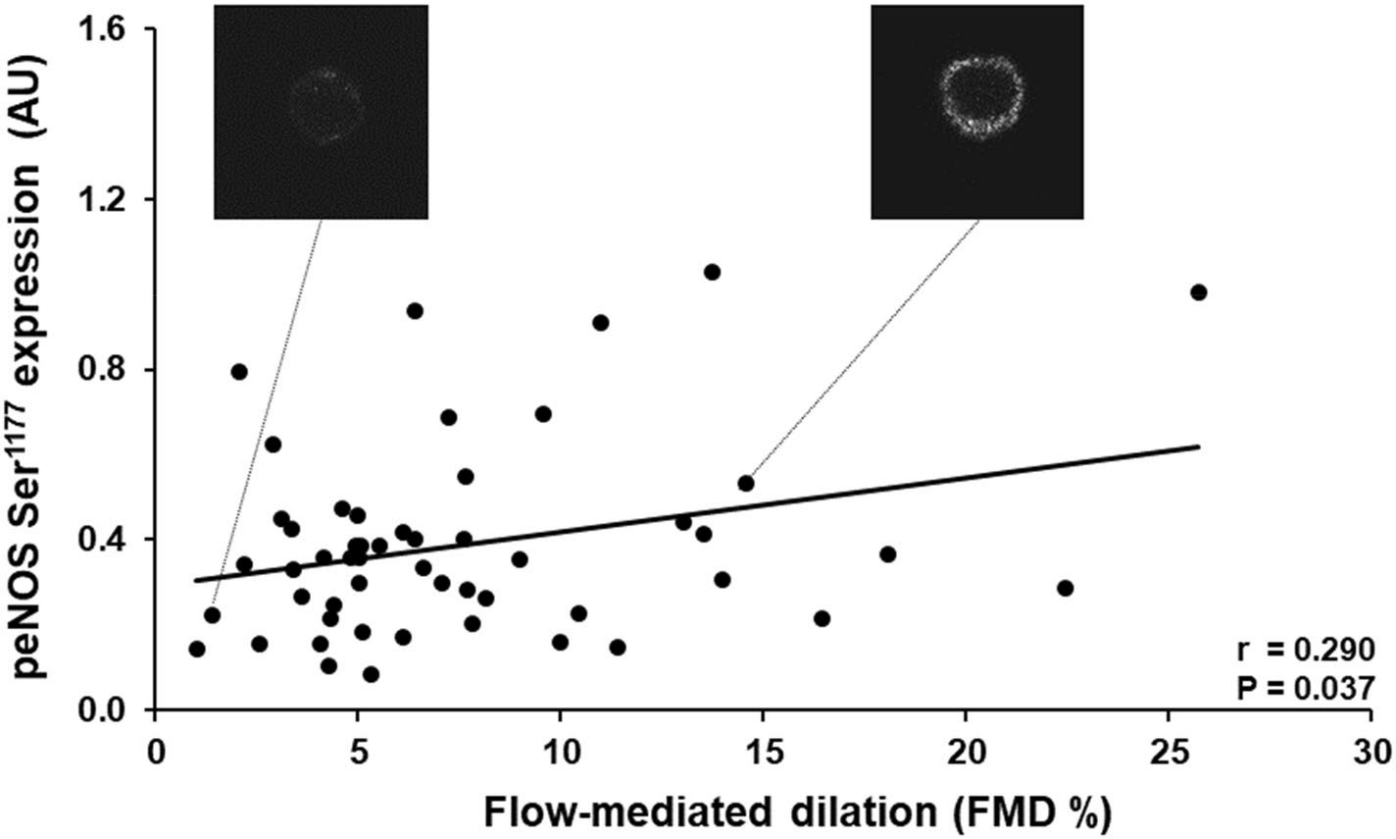
Positive correlation between flow-mediated dilation (FMD%) and eNOS Ser^1177^ phosphorylation (peNOS Ser^1177^) in endothelial cells (ECs) obtained from radial arteries of coronary artery disease patients. Representative images of the immunofluorescence images of peNOS Ser^1177^ from CAD patients with low and high FMD are shown. AU: arbitrary units EC protein expression data are reported as ratios to human umbilical vein endothelial cells (HUVEC) protein expression

**Fig. 2:**
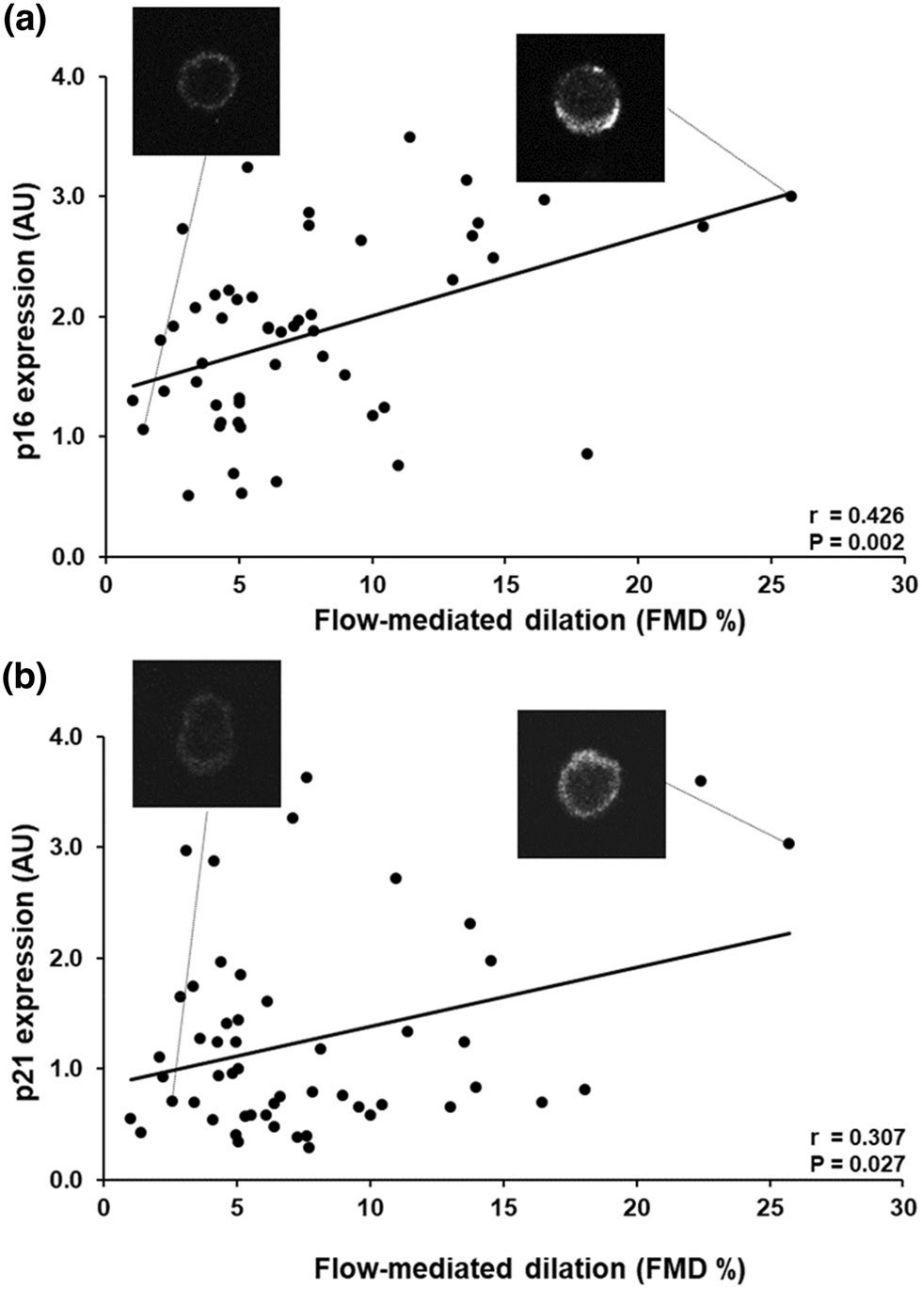
Positive correlation between flow-mediated dilation (FMD%) with p16 (**a**) and p21 (**b**) protein expression in endothelial cells (ECs) obtained from radial arteries of coronary artery disease patients. Representative images of the immunofluorescence images of p16 (**a**) and p21 (**b**) from CAD patients with low and high FMD are shown. *AU* arbitrary units EC protein expression data are reported as ratios to human umbilical vein endothelial cells (HUVEC) protein expression, *p16* cyclin-dependent kinase inhibitor 2A, *p21* cyclin-dependent kinase inhibitor 1

FMD was positively correlated with eNOS Ser^1177^ phosphorylation (*r* = 0.290, *P* = 0.037) (Fig. 1) and p16 (*r* = 0.426, *P* = 0.002) and p21 protein expression (*r* = 0.307 *P* = 0.027) (Fig. 2). When eNOS Ser^1177^ phosphorylation was normalized to eNOS content, the significant correlation was no longer present (*r* = 0.218, *P* = 0.121) (Table 2). Further, hierarchical multiple regression models suggested that p16 protein expression remained significantly associated with FMD (*P* = 0.008), despite accounting for MAP, BMI, sex and age. In contrast, p21 and eNOS Ser^1177^ phosphorylation associations with FMD appeared to be affected by the patients’ characteristics (*P* = 0.099 and *P* = 0.175, respectively), and the limiting factor in both cases was the MAP (*P* = 0.129 and *P* = 0.522). In contrast, BMI, sex and age appeared to not affect the associations between p21 and eNOS Ser^1177^ phosphorylation with FMD (*P* < 0.05). No significant association was observed between FMD and eNOS, NOX2, ET-1 and NFκB protein expression or nitrotyrosine abundance in patient ECs (Table 2). HE-mediated dilation was not correlated with any of the proteins examined (Table 2).

**Table 2:** Associations between flow-mediated dilation (FMD), vasodilation induced by handgrip exercise (HE%) and endothelial cell protein expression

	FMD (*N* = 54)	HE% (*N* = 46)
eNOS	*r* = − 0.039, *P* = 0.781	*r* = 0.227, *P* = 0.138
peNOS Ser^1177^	***r*** = 0.290, *P* = 0.037*	*r* = − 0.261, *P* = 0.087
peNOS Ser^1177^/eNOS	*r* = 0.218, *P* = 0.121	*r* = − 0.252, *P* = 0.099
ET-1	*r* = 0.177, *P* = 0.214	*r* = − 0.159, *P* = 0.308
p16	***r*** = 0.426, *P* = 0.002*	*r* = − 0.29, *P* = 0.852
p21	***r*** = 0.307, *P* = 0.027*	*r* = − 0.207, *P* = 0.178
p53	*r* = 0.108, *P* = 0.447	*r* = 0.049, *P* = 0.752
NT	*r* = 0.209, *P* = 0.140	*r* = 0.231, *P* = 0.136
NOX2	*r* = 0.177, *P* = 0.219	*r* = − 0.190, *P* = 0.222
NFκB	*r* = 0.105, *P* = 0.463	*r* = − 0.17, *P* = 0.915

*eNOS* endothelial nitric oxide synthase, *peNOS Ser 1177* phospo−eNOS Ser1177, *ET−1* endothelin−1, *p16* cyclin−dependent kinase inhibitor 2A, *p21* cyclin−dependent kinase inhibitor 1, *p53* tumor suppressor p53, *NT* nitrotyrosine, *NOX2* NADPH oxidase subunit NOX2, *NFκB* nuclear factor kappa−light−chain−enhancer of activated B cell

*Significant association with FMD (*P* < 0.05)

Pearson’s correlation coefficient (*r*) and *P* value are reported

### Relationship between endothelium-dependent dilation (FMD) and arterial response to exercise

There was no correlation between FMD and the percentage change in diameter (%HE) during handgrip exercise (*r* = − 0.015, *P* = 0.985).

### Associations among EC protein expression in CAD patients

#### eNOS Ser^1177^ phosphorylation is related to oxidative stress and senescence markers

**Fig. 3:**
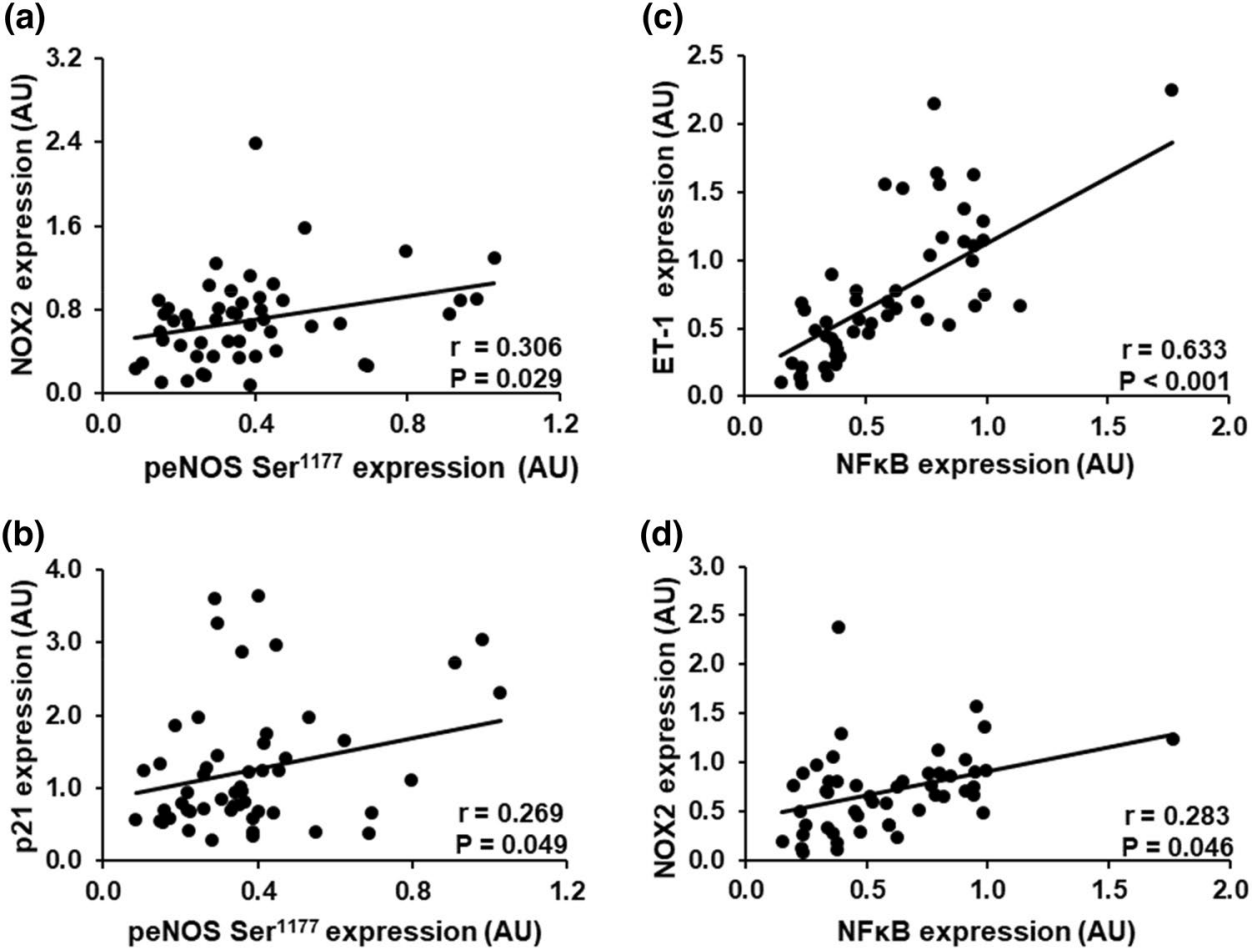
Positive relations between eNOS Ser^1177^ phosphorylation with NOX2 (**a**) and senescence marker p21 (**b**) of endothelial cells (ECs) obtained from radial artery of coronary artery disease patients. NFκB is correlated with ET-1 (**c**) and NOX2 (**d**). *AU* arbitrary units EC protein expression data are reported as ratios to human umbilical vein endothelial cells (HUVEC) protein expression; NOX2: NADPH oxidase subunit 2, *p21* cyclin-dependent kinase inhibitor 1, *NFκB* nuclear factor kappa-light-chain-enhancer of activated B cells, *ET-1* endothelin-1, *peNOS* phospho-endothelial nitric oxide synthase

There was a significant positive correlation between eNOS Ser^1177^ phosphorylation and NOX2 (*r* = 0.306, *P* = 0.029). When eNOS Ser^1177^ was normalized to eNOS content (peNOS Ser^1177^/eNOS), the correlation with NOX2 was close to significant (*r* = 0.271, *P* = 0.055). eNOS Ser^1177^ phosphorylation was also correlated with the expression of p21 (*r* = 0.269, *P* = 0.049) (Fig. 3). eNOS content and Ser^1177^ phosphorylation were not associated with any other EC protein (Table 3).

**Table 3:** Associations between endothelial cell protein expression in CAD patients (*n* = 54)

	eNOS	peNOS Ser^1177^	peNOS Ser^1177^/eNOS	ET-1	p16	p21	p53	NT	NOX2	NFκB
eNOS		*r* = 0.120 *P* = 0.387	***r* = − 0.446** ***P* = 0.001**	*r* = − 0.069 *P* = 0.623	*r* = − 0.229 *P* = 0.099	*r* = − 0.042 *P* = 0.763	*r* = 0.084 *P* = 0.544	*r* = − 0.124 *P* = 0.377	*r* = 0.005 *P* = 0.972	*r* = 0.074 *P* = 0.597
peNOS Ser^1177^	*r* = 0.120 *P* = 0.387		***r* = 0.706** ***p* < 0.001**	*r* = 0.222 *P* = 0.111	*r* = 0.075 *P* = 0.595	***r* = 0.269** ***P* = 0.049**	*r* = 0.067 *P* = 0.630	*r* = 0.044 *P* = 0.752	***r* = 0.306** ***P* = 0.029**	*r* = 0.115 *P* = 0.414
peNOS Ser^1177^/eNOS	***r* = − 0.446** ***P* = 0.001**	***r* = 0.706** ***p* < 0.001**		*r* = 0.182 *P* = 0.193	*r* = 0.079 *P* = 0.576	*r* = 0.202 *P* = 0.143	*r* = 0.114 *P* = 0.413	*r* = 0.091 *P* = 0.517	*r* = 0.271 *P* = 0.055	*r* = 0.083 *P* = 0.556
ET-1	*r* = − 0.069 *P* = 0.623	*r* = 0.222 *P* = 0.111	*r* = 0.182 *P* = 0.193		*r* = 0.080 *P* = 0.573	*r* = 0.020 *P* = 0.889	*r* = 0.041 *P* = 0.772	*r* = 0.169 *P* = 0.627	*r* = 0.157 *P* = 0.277	***r* = 0.633** ***P* < 0.001**
p16	*r* = − 0.229 *P* = 0.099	*r* = 0.075 *P* = 0.595	*r* = 0.079 *P* = 0.576	*r* = 0.080 *P* = 0.573		*r* = 0.106 *P* = 0.450	*r* = 0.225 *P* = 0.106	***r* = 0.638** ***P* < 0.001**	*r* = 0.132 *P* = 0.362	*r* = 0.042 *P* = 0.768
p21	*r* = 0.042 *P* = 0.763	***r* = 0.269** ***P* = 0.049**	*r* = 0.202 *P* = 0.143	*r* = 0.020 *P* = 0.889	*r* = 0.106 *P* = 0.450		***r* = 0.598** ***P* < 0.001**	***r* = 0.308** ***P* = 0.025**	***r* = 0.423** ***P* = 0.002**	*r* = − 0.065 *P* = 0.646
p53	*r* = 0.084 *P* = 0.544	*r* = 0.067 *P* = 0.630	*r* = 0.114 *P* = 0.413	*r* = 0.041 *P* = 0.772	*r* = 0.225 *P* = 0.106	***r* = 0.598** ***P* < 0.001**		***r* = 0.434** ***P* = 0.001**	***r* = 0.459** ***P* = 0.001**	*r* = 0.035 *P* = 0.806
NT	*r* = − 0.124 *P* = 0.377	*r* = 0.044 *P* = 0.752	*r* = 0.091 *P* = 0.517	*r* = 0.169 *P* = 0.627	***r* = 0.638** ***P* < 0.001**	***r* = 0.308** ***P* = 0.025**	***r* = 0.434** ***P* = 0.001**		*r* = 0.228 *P* = 0.102	*r* = 0.127 *P* = 0.370
NOX2	*r* = 0.005 *P* = 0.972	***r* = 0.306** ***P* = 0.029**	*r* = 0.271 *P* = 0.055	*r* = 0.157 *P* = 0.277	*r* = 0.132 *P* = 0.362	***r* = 0.423** ***P* = 0.002**	***r* = 0.459** ***P* = 0.001**	*r* = 0.228 *P* = 0.102		***r* = 0.283** ***P* = 0.046**
NFκB	*r* = 0.074 *P* = 0.597	*r* = 0.115 *P* = 0.414	*r* = 0.083 *P* = 0.556	***r* = 0.633** ***P* < 0.001**	*r* = 0.042 *P* = 0.768	*r* = − 0.065 *P* = 0.646	*r* = 0.035 *P* = 0.806	*r* = 0.127 *P* = 0.370	***r* = 0.283** ***P* = 0.046**	

*eNOS* endothelial nitric oxide synthase, *peNOS Ser1177* phospo−eNOS Ser1177, *ET−1* endothelin−1, *p16* cyclin−dependent kinase inhibitor 2A, *p21* cyclin−dependent kinase inhibitor 1, *p53* tumor suppressor p53, *NT* nitrotyrosine, *NOX2* NADPH oxidase subunit NOX2, *NFκB* nuclear factor kappa−light−chain−enhancer of activated B cells

Pearson correlation coefficient (*r*) and *P* value are reported

Significant associations (*P* < 0.05) were highlighted in bold

#### Relation between NFκB expression with vasoconstriction and oxidative stress

A strong positive correlation was observed between ET-1 and inflammatory marker NFκB (*r* = 0.633, *P* < 0.0001). A weak correlation was also reported between the expression of NOX2 and NFκB (*r* = 0.283, *P* = 0.046) (Fig. 3). NFκB was not associated with any other EC protein (Table 3).

#### Senescence markers are related to oxidative stress

**Fig. 4:**
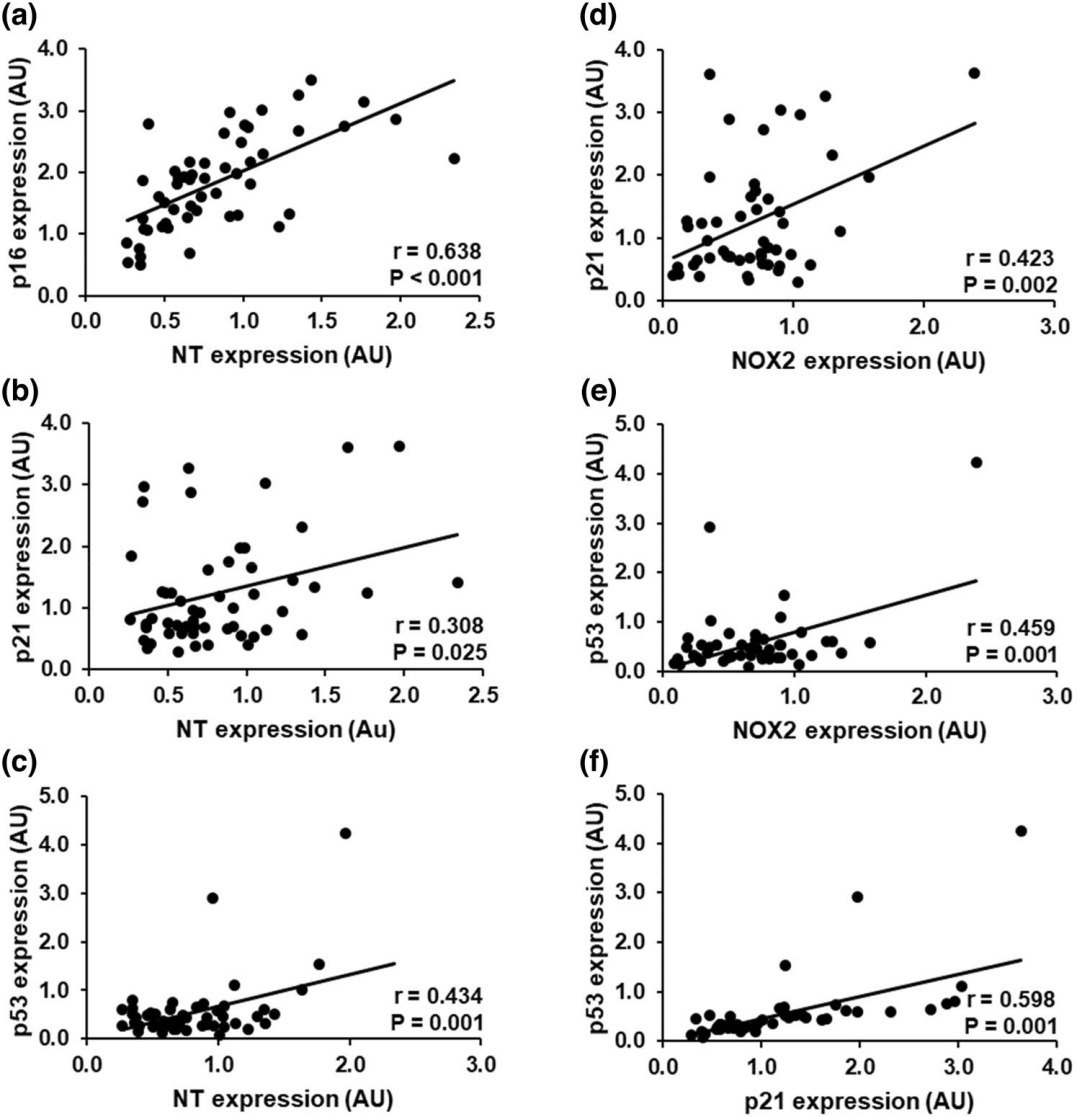
Positive correlations between senescence and oxidative stress expression of endothelial cells (ECs) obtained from radial artery of coronary artery disease patients. NT expression is related to p16 (**a**), p21 (**b**) and p53 (**c**), NOX2 expression is correlated to p21 (**d**) and p53 (**e**), and p21 expression is associated with p53 (**f**). *AU* arbitrary units EC protein expression data are reported as ratios to human umbilical vein endothelial cells (HUVEC) protein expression, *NT* nitrotyrosine, *p16* Cyclin-dependent kinase inhibitor 2A, *p21* cyclin-dependent kinase inhibitor 1, *p53* tumor protein 53, *NOX2* NADPH oxidase subunit 2

The expression of p16 (*r* = 0.638, *P* < 0.0001) p21 (*r* = 0.308, *P* = 0.025) and p53 (*r* = 0.434, *P* = 0.001) in ECs was positively correlated with the expression of nitrotyrosine. In addition, the expression of NOX2 was positively associated with the expression of p21(*r* = 0.423, *P* = 0.002) and p53 (*r* = 0.459, *P* = 0.001). Finally, there was a strong positive correlation between the expression of the senescence markers p21 and p53 (*r* = 0.598, *P* < 0.0001). These correlations are presented in Fig. 4. The senescence and oxidative stress markers were not associated with any other EC proteins (Table 3).

## Discussion

To our knowledge, the current study is the first to assess relationships between endothelium-dependent vasodilation and the expression of atherogenic risk-modulating proteins in patients with established CAD. Contrary to our hypothesis, the key novel findings were that: (1) endothelium-dependent dilation, assessed using FMD, was positively associated with eNOS Ser^1177^ phosphorylation seen in the isolated ECs and (2) markers of oxidative stress (NOX2 and nitrotyrosine) were not associated with FMD. In addition, positive associations were observed between FMD and the protein expression of the senescence markers p16 and p21. Interestingly, markers of inflammation (NFκB) and vasoconstriction (ET-1) were not associated with FMD. Moreover, no associations were observed between any of the proteins measured and arterial dilation in response to handgrip exercise. This finding together with the absence of correlation between FMD and exercise-induced vasodilation may suggest that different mechanisms underpin such responses.

### Association between endothelium-dependent dilation (FMD) and proteins involved in no production and scavenging

Previous work in individuals with CVD risk factors but without established CAD suggests that impaired endothelium-dependent dilation in humans is largely related to elevated oxidative stress (Donato et al. 2009; Seals et al. 2011; Gates et al. 2007; Lavi et al. 2008; O'Driscoll et al. 1999; O'Driscoll et al. 1997a, b; Cheetham et al. 2000) rather than impaired NO production (Donato et al. 2009; Pierce et al. 2011). In contrast with these observations in pre-clinical populations, we report that markers of oxidative stress (expression of NOX2 and nitrotyrosine content) were not associated with endothelium-dependent vasodilation in patients with established CAD. However, total eNOS Ser^1177^ phosphorylation, reflecting the overall activation of eNOS, was positively associated with endothelium-dependent dilation in these patients. Together with the lack of association between FMD and eNOS content or peNOS Ser1177/eNOS ratio may suggest that both lower eNOS content and ability to phosphorylate the available eNOS combine to reduce overall eNOS phosphorylation. The overall phosphorylation of eNOS is relevant (Hambrecht et al. 2003) as this appears to determine NO production and not the content or ability to phosphorylate the available eNOS (Mount et al. 2007).

Taken together the above evidence may indicate a difference in the regulation of endothelium-dependent dilation between patients with established CAD and pre-clinical subjects. Our data seem to suggest that further elevations in oxidative stress do not contribute to progressive declines in endothelium-dependent vasodilation in patients with diagnosed CAD. On the other hand, the activation of eNOS by Ser^1177^ phosphorylation becomes increasingly important to maintain NO production. In support of this, Hambrecht et al. (2003) reported a positive association between increased eNOS Ser^1177^ phosphorylation and endothelium-dependent dilation in the arteries of CAD patients. Furthermore, a number of drugs used to treat CVD and improve endothelium-dependent dilation act by stimulating eNOS Ser^1177^ phosphorylation (Huang 2009). Estrogens (Hisamoto et al. 2001), statins (Kureishi et al. 2000) and peroxisome proliferator activated receptors agonists (Aikawa et al. 2004) are all examples of such drugs.

In our ECs, we also demonstrated a positive association between endothelial eNOS Ser^1177^ phosphorylation and oxidative stress (NOX2 protein content). It has previously been shown that reactive oxygen species can modulate NO production by influencing eNOS activity (Li et al. 2014). Specifically, H_2_O_2_ has been shown to increase phosphorylation of eNOS at Ser^1177^ (Thomas et al. 2002). As such, the association between eNOS Ser^1177^ phosphorylation and NOX2 could reflect an attempt to increase NO production and overcome elevated NO scavenging by superoxide, as it has been hypothesized in aging (Donato et al. 2007, 2015).

### Association of endothelium-dependent dilation with markers of endothelial senescence

Cellular senescence, a stress-response resulting in irreversible growth arrest of a cell, is emerging as a potential driver of endothelial dysfunction and the development of atherosclerosis (Katsuumi et al. 2018). The accumulation of these cells alongside the pro-inflammatory and pro-oxidative phenotype of these cells have been proposed as mediators of the adverse effects of senescence (Childs et al. 2015; Minamino et al. 2002). Direct evidence associating senescence with endothelial function in humans is limited. However, recent work suggests that age-associated reductions in endothelium-dependent dilation were negatively associated with endothelial p53, p21 and p16 protein content (Rossman et al. 2017). In contrast to this, we report that FMD was positively associated with endothelial protein content of p21 and p16 in patients with established CAD. These positive associations were unexpected. Given the lack of human studies investigating vascular senescence in CAD, we cannot fully explain these observations. To explore the above paradoxical associations, we ran hierarchical multiple regressions accounting for patients’ characteristics. The results showed that endothelial senescence (at least p16 protein expression) was associated with higher endothelial function in these patients independently of individual characteristics. Mean arterial pressure appeared to influence the association between FMD and p21 expression. Endothelial (dys)function and arterial pressure are mechanistically (Konukoglu and Uzun 2017) and statistically related (Holder et al. 2021). Thus, it is difficult to differentiate the effect of arterial pressure on FMD, especially in our study which included uncontrolled hypertensives, controlled hypertensive and non-hypertensive patients. The prevailing hypothesis is that endothelial senescence leads to vascular dysfunction. However, studies in murine models have shown that knockout of p53 (Mercer et al. 2005) and p21 (Khanna 2009) resulted in greater severity of atherosclerotic lesions. As such, the role of senescence within the vasculature may depend on the stage of the progression of atherosclerosis (Childs et al. 2015, 2016). It is possible that senescence initially plays a protective role in atherosclerosis. Indeed, it has been proposed that senescent cells may delay the atherosclerotic process in the beginning as they prevent cell growth by activating cell arrest and/or perhaps apoptosis pathways (Mercer et al. 2005). Future studies should test the causal contribution of senescence at different stages of CAD pathology.

Markers of senescence (p53, p21 and p16) were associated with oxidative stress (EC nitrotyrosine abundance and NOX2 protein content). These associations are supported by previous work showing that oxidative stress leads to senescence through the activation of p53/p21 and p16 pathways (Bhayadia et al. 2016; Childs et al. 2015; Muñoz-Espín and Serrano 2014). Surprisingly, endothelial inflammation (NFκB) was not associated with any marker of senescence. Importantly, NFκB is known to initiate and maintain the senescence associated secretory phenotype via upregulating inflammatory mediators and reactive oxidative species (Salminen et al. 2012; Childs et al. 2015).

In addition, p21 protein content was associated with eNOS Ser^1177^ phosphorylation. This finding is also in contrast to previous data surrounding the role of senescence in ECs. In vitro data from human aortic ECs showed that senescence reduced eNOS protein content and activity (Matsushita et al. 2001). However, the positive association may provide a link between elevated expression of senescence markers and improved endothelium-dependent dilation, given that eNOS Ser^1177^ phosphorylation was also positively associated with endothelium-dependent dilation. The apparent disconnect between in vitro and in vivo data stresses the need for future human studies to evaluate the role of senescence at different stages of CAD development.

### Associations between inflammation (NFKB), oxidative stress (NOX2) and vasoconstrictor (ET-1)

NFκB is a key transcription factor in the regulation of pro-inflammatory markers (Kempe et al. 2005). Endothelial expression NFκB had a positive correlation with NOX2 protein content. This association suggests that NFκB may be an important link between inflammation and oxidative stress (Donato et al. 2009; Marchio et al. 2019). Indeed, previous work demonstrates the potential for NAD(P)H oxidase-dependent induction of NFκB (Clark and Valente 2004). In support of this, NFκB inhibition reduced NOX2 expression and improved endothelium-dependent dilation in obese subjects (Pierce et al. 2009). This suggests a vicious cycle between inflammation and oxidative stress.

The expression of the potent vasoconstrictor ET-1 had a strong positive correlation with NFκB expression. ET-1 has been proposed to affect endothelial function via inflammatory pathways (Bohm and Pernow 2007; Bohm et al. 2007). Cardiac overexpression of ET-1 in mice is associated with increased activation of NFκB (Yang et al. 2004). In turn, NFκB stimulates ET-1 expression (Bohm and Pernow 2007; Virdis and Schiffrin 2003). As such, our data further highlight the critical role of NFκB as a key regulator in the development of endothelial dysfunction in CAD.

### FMD does not predict arterial response to exercise

FMD was not correlated with the dilation induced during handgrip exercise. Exercise-induced dilation was also not associated with any of the atherogenic risk-modulating proteins assessed. Our team previously showed impaired FMD but preserved exercise-induced dilation in CAD patients following catheterization-induced damage (Tryfonos et al. 2020). This suggests different mechanisms may be responsible for dilation in these two tests. FMD is determined by a large and transient increase in shear stress. This is opposed to a more gradual increase in shear stress observed during exercise (Tremblay and Pyke 2018). FMD response is also known to be endothelium-dependent and mediated to a large extent though endothelial NO production (Green et al. 2014). In contrast, regulation of blood flow during exercise is a more complex process that involves a number of mechanisms (e.g., transmural pressure and vasoactive compounds) with multiple interactions and redundancy (Hellsten et al. 2012; Schrage et al. 2004, 2007; Green et al. 2017; Padilla et al. 2006). We believe the lack of association between FMD and exercise response in CAD patients add weight to the argument of different mechanisms underlined among these physiological responses.

A limitation of this study is that vascular measurements were taken on the day of catheterization. As such, although guidelines for FMD were followed (Thijssen et al. 2019), patients continued their medications as instructed by their consultant. Such medications could have affected the in vivo assessments of vascular function but are unlikely to affect the protein expression of ECs. Previous studies in CAD patients suggest that our radial FMD data are within the normal range (Dawson et al. 2010, 2012). Handgrip exercise was conducted 10 min after FMD assessment. To our knowledge, the influence of prior FMD assessment on response to handgrip exercise has not been established. Therefore, it is possible that the response to the handgrip exercise was influenced by the earlier hyperaemic stimulus. However, previous work has shown that FMD response was not influenced by earlier FMD assessment when the vessel was allowed to rest for ~ 5 min (Barton et al. 2011). Finally, the current study only measured total EC expression of NFκB p65. Given that translocation of NFκB to the nucleus is required to exert its actions on gene transcription, future work should expand this preliminary assessment and investigate nuclear abundance of NFκB p65 (Donato et al. 2008).

## Summary and conclusions

The results of this study provide new insight into the molecular events underlying the continued development of endothelial dysfunction in patients with established CAD. In contrast to our hypothesis, we demonstrated that FMD in patients with established CAD was positively associated with markers of NO production (eNOS Ser^1177^ phosphorylation). Again, contrary to our hypothesis, FMD was not associated with markers of oxidative stress and inflammation (nitrotyrosine abundance and protein content of NOX2, NFκB and ET-1). Comparing to previous studies, our data suggest that there is a difference in the regulation of endothelium-dependent vasodilation between patients with established CAD and those without a clinical diagnosis. Our data in the isolated ECs suggest that patients with established CAD manage to maintain vasodilation by activating eNOS at Ser^1177^ residue. Given the association between senescence markers and FMD, future work should examine the role of senescence in the progression of CAD.

## Data Availability

The datasets generated during and/or analyzed during the current study are available from the corresponding author on reasonable request.

## Abbreviations

BP: Blood pressure
CAD: Coronary artery disease
CVD: Cardiovascular disease
EC(s): Endothelial cell(s)
eNOS: Endothelial nitric oxide synthase
ET-1: Endothelin 1
FMD: Flow-mediated dilation
HE: Handgrip exercise
HR: Heart rate
HUVEC: Human umbilical vein endothelial cell
LHCH: Liverpool Heart and Chest Hospital
MVC: Maximal voluntary contraction
NFκB: Nuclear factor kappa-light-chain-enhancer of activated B cell
NO: Nitric oxide
NOX-2: NADPH oxidase subunit NOX2
NT: Nitrotyrosine
PCI: Percutaneous coronary intervention
peNOS Ser 1177: Phospo-eNOS Ser1177
PTCA: Percutaneous transluminal coronary angiography
p16: Cyclin-dependent kinase inhibitor 2A
p21: Cyclin-dependent kinase inhibitor 1
p53: Tumor suppressor p53
VE-cadherin: Vascular endothelial cadherin
